# Pentavalent Antimonials Combined with Other Therapeutic Alternatives for the Treatment of Cutaneous and Mucocutaneous Leishmaniasis: A Systematic Review

**DOI:** 10.1155/2018/9014726

**Published:** 2018-12-24

**Authors:** Taisa Rocha Navasconi Berbert, Tatiane França Perles de Mello, Priscila Wolf Nassif, Camila Alves Mota, Aline Verzignassi Silveira, Giovana Chiqueto Duarte, Izabel Galhardo Demarchi, Sandra Mara Alessi Aristides, Maria Valdrinez Campana Lonardoni, Jorge Juarez Vieira Teixeira, Thaís Gomes Verziganassi Silveira

**Affiliations:** ^1^Graduate Program in Health Sciences, State University Maringá, Avenida Colombo, 5790 Jardim Universitário, 87020-900, Maringá, PR, Brazil; ^2^Graduate Program in Bioscience and Physiopathology, State University Maringá, Avenida Colombo, 5790 Jardim Universitário, 87020-900 Maringá, PR, Brazil; ^3^Medical Residency, Santa Casa de São Paulo, R. Dr. Cesário Mota Júnior, 112 Vila Buarque, 01221-900 São Paulo, SP, Brazil; ^4^Undergraduation Course in Medicine, State University Maringa, Avenida Colombo, 5790 Jardim Universitário, 87020-900 Maringá, PR, Brazil; ^5^Department of Clinical Analysis and Biomedicine, State University Maringa, Avenida Colombo, 5790 Jardim Universitário, 87020-900 Maringá, PR, Brazil

## Abstract

The first choice drugs for the treatment of cutaneous and mucocutaneous leishmaniasis are pentavalent antimonials, sodium stibogluconate, or meglumine antimoniate. However, the treatment with these drugs is expensive, can cause serious adverse effects, and is not always effective. The combination of two drugs by different routes or the combination of an alternative therapy with systemic therapy can increase the efficacy and decrease the collateral effects caused by the reference drugs. In this systematic review we investigated publications that described a combination of nonconventional treatment for cutaneous and mucocutaneous with pentavalent antimonials. A literature review was performed in the databases Web of Knowledge and PubMed in the period from 01^st^ of December 2004 to 01^st^ of June 2017, according to Prisma statement. Only clinical trials involving the treatment for cutaneous or mucocutaneous leishmaniasis, in English, and with available abstract were added. Other types of publications, such as reviews, case reports, comments to the editor, letters, interviews, guidelines, and errata, were excluded. Sixteen articles were selected and the pentavalent antimonials were administered in combination with pentoxifylline, granulocyte macrophage colony-stimulating factor, imiquimod, intralesional sodium stibogluconate, ketoconazole, silver-containing polyester dressing, lyophilized LEISH-F1 protein, cryotherapy, topical honey, and omeprazole. In general, the combined therapy resulted in high rates of clinical cure and when relapse or recurrence was reported, it was higher in the groups treated with pentavalent antimonials alone. The majority of the articles included in this review showed that cure rate ranged from 70 to 100% in patients treated with the combinations. Serious adverse effects were not observed in patients treated with drugs combination. The combination of other drugs or treatment modalities with pentavalent antimonials has proved to be effective for cutaneous and mucocutaneous leishmaniasis and for most seemed to be safe. However, new randomized, controlled, and multicentric clinical trials with more robust samples should be performed, especially the combination with immunomodulators.

## 1. Introduction

Leishmaniasis is an important zoonosis around the world, being reported that about 20,000 to 30,000 deaths occur annually as a consequence of the disease [1]. The most frequent form is cutaneous leishmaniasis (CL), which is present in several countries, mainly in the Americas, the Mediterranean basin, the Middle East, and Central Asia. An annual occurrence of 0.6 to 1.0 million new cases is estimated [2] and around 399 million of people are at risk of infection in 11 high-burden countries [1].

The pentavalent antimonials, sodium stibogluconate or meglumine antimoniate, are drugs commonly used to treat cutaneous and mucocutaneous leishmaniasis. However, the treatment with these drugs is expensive and can cause serious adverse effects, such as cardiac toxicity and elevation in the levels of hepatic enzymes [3–5], and, sometimes, it is ineffective or presents low cure rates [6, 7]. Amphotericin B, pentamidine, fluconazole, and miltefosine can be used as second choice drugs, but they also exhibit toxicity. Moreover, the efficacy of the treatment also depends on the* Leishmania* species involved in the infection, since some species are more resistant to some drugs [6].

Local therapies, such as cryotherapy, CO_2_ laser, thermotherapy, and photodynamic therapy, are alternatives to conventional drugs, since they are less toxic to the patient and the main adverse effects are restricted to the site of application [8–13]. However, the exclusive use of local therapy is controversial, since some New World species can lead to mucosal leishmaniasis after primarily cutaneous lesions [3].

The combination of two drugs or the combination of a local therapy with systemic therapy can be an alternative to increase the efficacy of local therapy and may decrease the collateral effects caused by the reference drugs. Some studies have evaluated the efficacy of this type of combination [14–17], being necessary prospective and multicenter studies for safer evidence. Our central question was evaluated if the combination of an alternative therapy with meglumine antimoniate presents more efficiency that only meglumine antimoniate in the treatment of cutaneous and mucocutaneous leishmaniasis. In this sense, we investigated published articles that used the combination of an alternative therapy with pentavalent antimonials in the treatment of cutaneous and mucocutaneous leishmaniasis through systematic review.

## 2. Methodology

### 2.1. Literature Search

A literature review was performed in the databases Web of Knowledge and PubMed, considering the period from 01^st^ December 2004 to 01^st^ June 2017 according to Prisma statement [18]. The screening of the titles and abstracts was performed by researchers (TRNB, CAM, PWN, TFPM, GCD and AVS). The MeSH (Medical Subject Headings) terms, strategy used for the search on PubMed, were also selected by these researchers based on publications on the topic at PubMed. Any disagreements were decided by consensus. The MeSH terms were validated by two experts (JVT and TGVS) and were divided into two groups: Group 1 “Antiprotozoal Agents” OR “Combined Modality Therapy” OR “Drug Therapy, Combination” OR “Treatment Outcome” OR “Amphotericin B” OR “Meglumine” OR “Protozoan Vaccines” OR “Organometallic Compounds” OR “Antimony Sodium Gluconate” OR “Antimony” OR “Pentamidine” OR “Anti-Infective Agents” OR “Medication Therapy Management” OR “Complementary Therapies”; AND Group 2 “Leishmaniasis” OR “Leishmania”. The research in the Web of Knowledge database was carried out by topic, which ensures good sensitivity.

### 2.2. Inclusion, Exclusion Criteria, and Studies Selection

Articles that describe a combination of therapeutic alternatives with pentavalent antimonials for cutaneous or mucocutaneous leishmaniasis were included in this review. Only original clinical trials, in English and with abstract available, were added. Other types of publications (reviews, case reports, comments to the editor, letters, interviews, guidelines, and errata) were excluded. After the search the papers initially selected were analyzed by the researchers of group 1 (TRNB, CAM, PWN, TFPM, GCD, and AVS) and disagreements about inclusion or exclusion of articles were decided by consensus. To increase the search sensitivity, the researchers in group 1 checked all references from the selected publications to retrieve other unidentified publications in the other phases of the search. The validation of selected articles was performed by four independent evaluators of group 2 (TGVS, MVCL, SMAA, and IGD).

### 2.3. Data Extraction

The structure of the topics to compose the tables was organized by researchers from group 1 with the support of two experts (TGVS and JVT): Table 1 (study, area country, study design, period of study, age range or mean in years, gender, clinical forms, patients enrolled, leishmaniasis diagnosis, and statistics); Table 2 (study,* Leishmania* species, treatment, patients at the end, percentage of clinically healed patients or lesions, percentage of therapy failure, and percentage of relapse or recurrence); Table 3 (treatment, side effects percentage, and study source); and Table 4 (treatment, dose, route of administration, time efficacy, safety, practice/clinical implications, and study source). The tables were completed by researchers in group 1 and then checked by researchers from group 2.

## 3. Results

**Figure 1:**
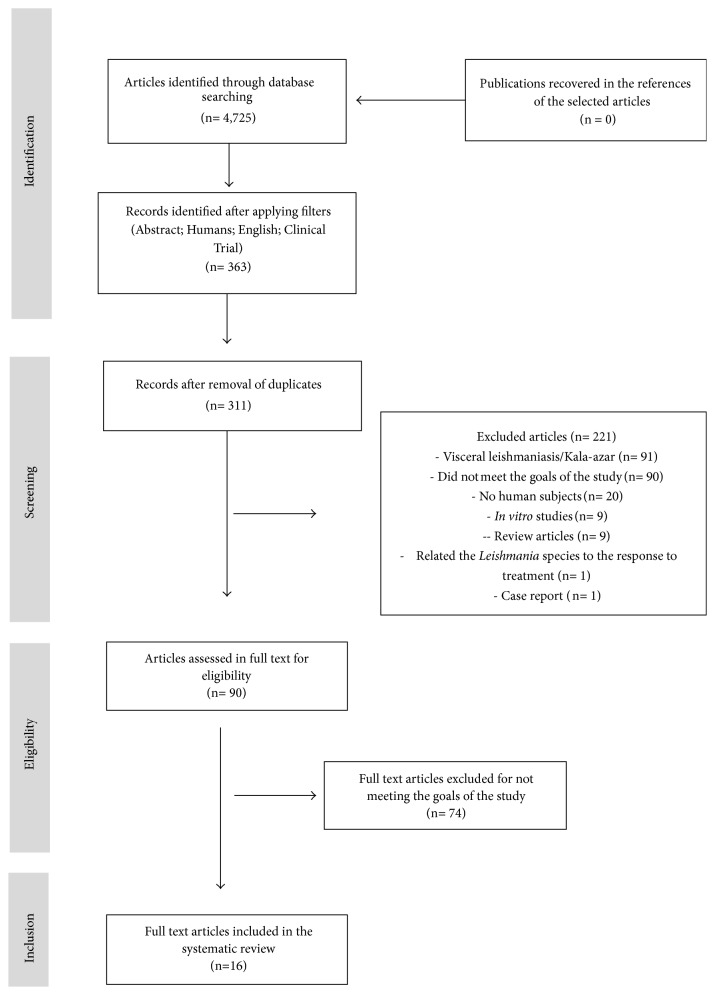
Flow diagram of study selection for the systematic review.

Based on the inclusion criteria defined by consensus, 16 articles were selected, being from Iran (6), Peru (4), Brazil (4), Yemen (1), and Afghanistan (1) (see Figure 1). In all, 1,302 patients aged between 1 and 87 years were involved in the studies, with cutaneous or mucocutaneous leishmaniasis, being predominant the cutaneous form of the disease. The most reported species of* Leishmania* were* L. braziliensis*,* L. tropica,* and* L. major *(Table 1).

In the selected articles, pentavalent antimonials were administered in combination with different drugs or treatment modalities, which were pentoxifylline; granulocyte macrophage colony-stimulating factor; imiquimod; intralesional sodium stibogluconate; ketoconazole; nonsilver-containing polyester dressing; silver-containing polyester dressing; lyophilized LEISH-F1 protein; cryotherapy, topical honey, and omeprazole.

Among the patients involved in the studies, 92.0% (1199/1302) ended the treatment, of which 48.0% (575/1199) underwent a combination treatment (antimonial pentavalent plus other treatment) and the remaining 52.0% (624/1199) were treated only with pentavalent antimonials or other treatment modalities (Table 2). Most of them had not undergone previous treatments.

The combination of drugs revealed high rates of clinical cure among the groups treated with drug combination. Two papers reported a cure rate of 100% in these groups (Almeida et al. 2005 [19]; Arevalo et al. 2007 [20]), while 8 authors reported 70-94% cure in the groups treated with combinations (El-Sayed and Anwar 2010 [21]; Llanos Cuentas et al. 2010 [22]; Machado et al. 2007 [15]; Meymand et al. 2011 [10]; Miranda-Verastegui et al. 2005 [23]; Miranda-Verastegui et al. 2009 [24]; Nascimento et al. 2010 [25]; Nilforoushzadeh et al. 2008 [26]). The other authors reported cure rates below 70% and ranged from 36.4% to 66.7%. The lowest cure rate was (36.4%) in the combination of IL-MA+ silver PD (Khatami et al. 2013 [27]) (Table 2).

Among the combinations, those with 100% of cure rate were meglumine antimoniate (MA) plus granulocyte macrophage colony-stimulating factor (GM-CSF) (Almeida et al. 2010) and meglumine antimoniate plus imiquimod (Arevalo et al. 2007). The other combinations that resulted in 70-94% of cure were the combinations of sodium stibogluconate (SSG) plus LEISH-F1 + MPL-SE (94%) (Llanos Cuentas et al. 2010); intralesional sodium stibogluconate plus ketoconazole (90%) (El-Sayed and Anwar 2010); meglumine antimoniate plus omeprazole (89%) (Nilforoushzadeh et al. 2008); MA and pentoxifylline (82%) (Machado et al. 2007); meglumine antimoniate plus LEISH-F1 + MPL-SE (80%) (Nascimento et al. 2010); intralesional sodium stibogluconate plus cryotherapy (78%) (Meymand et al. 2011); sodium stibogluconate plus imiquimod (75%) (Miranda-Verastegui et al., 2009); and meglumine antimoniate plus imiquimod (72%) (Miranda-Verastegui et al. 2005). It is important to note that most combinations that showed high cure rates (70-100%) were combinations of pentavalent antimonial with some immunomodulators.

Relapse or recurrence, when reported, was higher in the groups treated with pentavalent antimonial alone and varied from 0 to 38% (Llanos Cuentas et al., 2010; Nascimento et al., 2010 [22, 25]). For the associated groups, only four associations presented relapse or recurrence, and these rates ranged from 0 to 11.1% (Firooz et al. 2006; Van-Thiel et al. 2010 [28, 29]) (Table 2).

No serious adverse effects were observed in patients treated with the drugs combination. For the combination of imiquimod and meglumine antimoniate, adverse effects were locally limited, being the most reported pruritus/itching, erythema, and edema. For the combination of imiquimod with sodium stibogluconate, the same was observed. Only Miranda-Verastegui et al. (2005) [23] reported elevated liver enzyme levels.

In relation to granulocyte macrophage-stimulating factor, there were no reports of side effects. With lyophilized LEISH-F1 protein in association to meglumine antimoniate, the observed side effects were induration, erythema, and tenderness; in combination with sodium stibogluconate, the presence of induration, erythema, and tenderness sites was reported, in addition to headache pyrexia and systemic malaise. The common adverse effects of the use of meglumine antimoniate and sodium stibogluconate were also observed.

To the combination of meglumine antimoniate and pentoxifylline, the common adverse effects, described in two studies, were nausea, arthralgia, dizziness, pain, and diarrhea.

In the use of intralesional sodium stibogluconate, alone or in association with other medicinal products, secondary infection, pain and swelling at injection site, and lymphatic involvement were observed. The pentavalent intralesional antimonials also showed adverse effects related to the application site, such as pain, pruritus/itching, and edema.

Intralesional sodium stibogluconate, when associated with cryotherapy, resulted in secondary infection and lymphatic involvement, in addition to the inherent symptoms of intralesional application of stibogluconate already mentioned. Meglumine antimoniate combined with silver PD presented only itching, burning, and edema, in contrast to when combined with topical honey, in which only dermatitis, caused by honey, was reported. Cryotherapy combined with meglumine antimoniate had only local adverse effects such as hyperpigmentation plus trivial scar, atrophic scar, and hypopigmentation plus trivial scar (Table 3).

Each of the combinations was classified according to their efficacy (efficacious/likely efficacious/not efficacious) and the clinical implications (investigational/clinically useful/possibly useful) [30].

In this context, imiquimod associated with meglumine antimoniate (Miranda-Verastegui et al. 2005 [23]) and stibogluconate (Miranda-Verastegui et al. 2009 [24]) and cryotherapy-associated stibogluconate (Van-Thiel et al. 2010 [29]) were classified as clinically useful and with acceptable risk without specialized monitoring. The meglumine antimoniate associated with pentoxifylline (Machado et al. 2007) were classified as clinically useful and with acceptable risk with specialized monitoring (Machado et al. 2007 [15]). On the other hand the combination of meglumine antimoniate associated with pentoxifylline performed by Brito et al. 2017 [31] to treat cutaneous leishmaniasis caused by* Leishmania braziliensis* was classified as not efficacious and not useful. Meglumine antimoniate associated with omeprazole (Nilforoushzadeh et al. 2008 [26]) was classified as clinically useful and with acceptable risk with specialized monitoring.

Some combinations have been classified as possibly useful with acceptable risks without specialized monitoring, such as cryotherapy combined with meglumine antimoniate (Farajzadeh et al. 2015 [32]) and the intralesional meglumine antimoniate with cryotherapy (Meymandi et al. 2011 [10]). The combination LEISH-F1 + MPL-SE plus meglumine antimoniate (Nascimento et al. 2010 [25]) and sodium stibogluconate with ketoconazole (EL-Sayed and Anwar 2010 [21]) was classified as possibly useful and with an acceptable risk with specialized monitoring.

GM-CSF plus meglumine antimoniate (Almeida et al. 2005 [19]) was still classified as investigational and with acceptable risk without specialized monitoring, while other combinations were classified as investigational, but with acceptable risk with specialized monitoring, such as: imiquimod plus meglumine antimoniate (Arevalo et al., 2007 [20]), Leish-F1+ MPLE-SE plus sodium stibogluconate (Llanos Cuentas et al. 2010 [22]), meglumine antimoniate combined with silver PD (Khatami et al. 2013 [27]), and imiquimod plus meglumine antimoniate (Firooz et al. 2006 [28]). The evidence provided by the study with the combination of intralesional meglumine antimoniate and topical honey was insufficient to classify this combination in relation to safety (Table 4).

Regarding effectiveness, only three combinations were classified as noneffective: intralesional meglumine antimoniate associated with topical honey performed by Nilforoushzadeh et al. (2007) [33], intralesional meglumine antimoniate associated with silver PD tested by Khatami et al. (2013) [27], and pentoxifylline plus meglumine antimoniate performed by Brito et al. (2017). The other combinations were classified as “efficacious” or “likely efficacious”.

## 4. Discussion

In this review, we saw that the majority of the combinations resulted in an elevated cure rate. Relapse or recurrence, when reported, were higher in the groups treated with the isolated drugs than in the ones treated with the drugs combination. These findings indicate that the combinations with pentavalent antimonials were more efficacious to prevent relapse or recurrence. Several authors have demonstrated that the combination of some drugs with pentavalent antimonial showed a higher percentage of cure.

### 4.1. Pentavalent Antimonials

Pentavalent antimonials are considered the first line drugs to treat CL, but they have collateral effects and, in some cases, low cure rate. According to a systematic review by Tuon et al. (2008) [34], meglumine antimoniate (MA), in the recommended dose (20 mg/kg/day), presents an average cure of 76.5%. However, among the studies evaluated by Tuon et al. (2008) [34] and other studies, meglumine antimoniate (20 mg/kg/day) cure rates are quite variable: 40.4% [7, 16], 56.9% [35], 69.4% [7], 79% [36], 84% [5], 85% [37], and 100% [38, 39].

For sodium stibogluconate (SSG), the cure rate shown by Tuon et al. (2008) [34] was of 75.5% in different dosages, with a maximum dose of 20 mg/kg/day. However, the efficacy for this pentavalent antimonial is also variable, being reported rates of 53% [24], 56% [40], 70% [41], and 100% [22, 42].

It is known that the use of systemic meglumine antimoniate can be lead to serious adverse effects, so the application in the lesion site showed to be an efficacious and more secure alternative to treat CL. Some authors have demonstrated that the intralesional MA is as effective as the systemic MA and had few adverse effects [43–45]. It is important to note that, unlike in the articles included in this study, Vasconcellos et al. (2014) [46] reported that one patient presented eczema after the treatment with intralesional meglumine antimoniate. After use of oral dexchlorpheniramine, eczema and ulcer receded. Thus, the administration of intralesional MA must be carefully conducted, especially due to the possibility of occurring hypersensitivity.

For the SSG, the intralesional application has also shown good results [47, 48]. The application twice a week is well tolerated and the lesions healed faster than only once a week [49].

### 4.2. Granulocyte Macrophage Colony-Stimulating Factor (GM-CSF)

The granulocyte macrophage colony-stimulating factor (GM-CSF) acts in the recruitment of monocytes and neutrophils. It is produced by a wide range of cells such as macrophages, neutrophils, dendritic cells, T cells, eosinophils, fibroblasts and endothelial cells. It is also believed that it promotes the differentiation of the macrophages to a proinflammatory phenotype [50].

In view of its role in the recruitment of different types of cells, GM-CSF has been investigated for the CL treatment. In their study, Almeida et al. (2005) [19] evaluated the topical use of GM-CSF (10 *μ*g/mL) in combination with the meglumine antimoniate (20 mg/kg/day) and showed that 60% of the patients were clinically healed 50 days after the treatment start, and the remaining 40% were cured 120 days after the beginning of the treatment. Similar results were found by Santos et al. (2004) [51], when they use this combination. On the other hand, among the patients treated only with meglumine antimoniate, just 20% were clinically healed at 45 days after the start of treatment, and 100% of the patients were cured after 256 days.

In a previous study, Almeida et al. (1999) [52] showed that clinical cure in patients treated with the combination of pentavalent antimonial and GM-CSF was faster than in the control group that was treated with pentavalent antimonial alone. Possibly the factor that contributed for the quick cure associated by GM-CSF was the modulation of the immunologic balance, by inducing differentiation for the Th1 subtype [52–54] and activation of macrophages to kill* Leishmania* [55].

GM-CSF combined with pentavalent antimonial can be an alternative to treat CL, since the risk inherent to this combination is acceptable and its use deserves to be greatly investigated.

### 4.3. Imiquimod

Imiquimod is an immunomodulator that was first approved to treat genital and perianal warts and then to treat actinic keratosis.

Imiquimod stimulates the immune system in different ways. It is believed that imiquimod is an agonist of the tool like receptors 7 and 8, so the stimulation of these receptors leads to the synthesis of different inflammatory mediators, such as INF-*α*, TNF-*α*, interleukins 1, 6, 8, 10 and 12, granulocyte colony -stimulating factor and granulocyte macrophage colony-stimulating factor [56–58]. In addition, the use of imiquimod also indirectly contributes to the immune response acquired, through the induction of Th1 type cytokines, such as INF-*ϒ* [58, 59]. The induction of INF-*ϒ* an IL-12 production induces to Th1 differentiation and it is important in the control of CL.

Imiquimod has been investigated in the treatment of CL and its efficacy is controversial. Arevalo et al. (2007) [20] and Seeberger et al. (2003) [60] showed no efficacy in the use of imiquimod alone. In combination with pentavalent antimonials, imiquimod can be an adjuvant; moreover, the success in treatment with imiquimod is directly related to the concentration used. Only at the concentration of 7.5% imiquimod combined with meglumine antimoniate appears to be more effective than the antimonate alone [20]. Authors that administered imiquimod at 5% in combination with meglumine antimoniate observed that the efficacy was similar to that of patients treated with meglumine antimoniate alone [23, 28].

However, when Miranda-Verastegui et al. (2009) [24] used imiquimod 5% combined with sodium stibogluconate, the combination was more effective than sodium stibogluconate alone.

Meymandi et al. (2011) [61] showed the combination of intralesional meglumine antimoniate and imiquimod as beneficial its resulted in a decrease in parasitic load, an increase in lymphocyte numbers, and a decrease in histiocyte aggregation in the lesion site. In addition, they observed that imiquimod alone was also ineffective.

Imiquimod appears to be a good adjuvant for pentavalent antimonial when used in the appropriate concentration. The risk involved in its use is acceptable. More evidence is needed to strengthen its application in clinical practice.

### 4.4. Silver-Containing Polyester Dressing

The silver-containing polyester dressing (silver PD) is composed of hydrophobic polyamide netting with silver-coated fibers. Silver PD differs from each other by the way silver is incorporated and how it is liberated in the lesion. It is known that silver has antimicrobial activity in solutions, but it does not differentiate at pathogens from the other cells, such as fibroblast and keratinocytes [62, 63].

Clinical trials using silver PD to treat CL are scarce. In this review, only one study used silver PD with this aim. No efficacy in silver PD was shown, not even combined with intralesional meglumine in the treatment of CL [27]. In this study, silver PD Atrauman Ag® by Hartmann was used.

As mentioned before, silver can cause the death of human cells [63]. However, according to the manufacturer of the Atrauman Ag®, a higher concentration of silver is needed to lead to the death of human cells and, specifically in the case of Atrauman Ag®, the release of silver is small. Moreover this dressing released silver only when in contact with bacteria and no negative influence of the silver ions was exercised in human cells [64]. Since amastigote forms are phagocytosed by macrophages, they remaining and multiplying. The silver released by the dressing, for being in small quantities, may not be able to reach the amastigotes phagocytosed.

There are some inherent characteristics of polyester dressing that influence in their activity, such as their capacity in the release of silver [65]. Besides that, the compounds binding to silver can contribute to this activity.

The use of silver PD isolated or in combination with pentavalent antimonial needs to be further investigated due to the scarcity of studies that used silver PD to treat CL and the several factors that can influence its efficacy.

### 4.5. LEISH-F1+MPL-SE

LEISH-F1+MPL-SE was the first candidate vaccine for entry in clinical trials. It was composed by recombinant fusion protein Leish-111f and an adjuvant in an oil-water emulsion (monophosphoryl lipid A - MPL). MPL is a TLR4 agonist, safely used in other vaccines, such as hepatitis [66].

Authors demonstrated that LEISH-F1+MPL-SE was safe, immunogenic, and effective in inducing the production of IgG antibodies, INF-*ϒ*, and other cytokines in humans and mice [67–69].

In the two articles included in this review, LEISH-F1+MPL-SE was tested in combination with SSG or meglumine antimoniate in the treatment of CL. One of these Llanos Cuentas et al. (2010) [22] observed similar clinically cure in both groups; however in addition, relapse or recurrence did not occur in the combination groups. The stimulation of the immune response was greater in the LEISH-F1+MPL-SE group than in the SSG group, a fact that may have contributed to the absence of recurrences.

Nascimento et al. (2010) [25], on the other hand, observed a greater clinical cure rate (80%) in the group treated with the combination of LEISH-F1+MPL-SE and meglumine antimoniate than in the groups treated with meglumine antimoniate alone (38%) or the adjuvant MPL-SE alone (50%).

LEISH-F1+MPL-SE in combination with pentavalent antimonials can be useful to treat CL, mainly because this combination appears to decrease recurrences observed with pentavalent antimony alone. The risks related to its use are acceptable therefore its use should be better explored.

### 4.6. Topical Honey

Honey was used, many years ago to treat several types of lesions, but there is no consensus on its effectiveness in lesion healing. In relation to CL, there are few data on the use of honey for its treatment.

It is well established that honey has an antimicrobial action, which can act on tissues, contributing to their repair [70], and also on the immune system, having both proinflammatory and anti-inflammatory action [71].

FDA has already approved some honey-based products with different clinical indications, but some authors remain cautious regarding its clinical use for lesion healing. Jull et al. (2013) [72], in a review about the use of topical honey in the treatment of wounds, concluded that honey may delay the time of wound healing in some types of wounds, such as CL and deep burns, but it is good for moderate burns. Still, in their opinion, more clinical studies are needed to guide the use of honey in clinical practice in other types of wounds than moderate burns.

In the same line Saikaly and Khachemoune (2017) [73] concluded in their study that the use of honey seems to be beneficial to wound healing in some types of lesions and that new technologies have contributed to the understanding of the action mechanisms of honey. However, more evidence is still needed to elucidate precisely the results obtained with the use of honey.

The combination of topical honey with IL-MA to treat CL was tested by Nilforoushzadeh et al. (2007) [33] and did not show efficacy. In this study, gauze soaked in honey was used, not being mentioned the type of honey used. It is known that there are different types of honey of different constitution and that, therefore, they may have different properties [71]. The choice of dressing must also be taken into consideration, as one should choose the dressing most appropriate for the wound to be treated [70].

There are several factors related to honey that should be taken into account, such as honey type and composition, as well as the best form of application, and it deserves to be better evaluated in order to be combined with pentavalent antimonials in the treatment of CL.

### 4.7. Omeprazole

Omeprazole is a drug used to treat peptic ulcer disease, due to its interference with the stomach pH. Omeprazole acts by inhibiting the human gastric K^+^, H^+^-ATPase enzyme, resulting in the disruption of acid secretion [74].

In the intracellular environment, omeprazole accumulates in the lysosomes, in the same place that the amastigotes in the macrophages. Jiang et al. (2002) [75] showed that omeprazole inhibits the K^+^, H^+^-ATPase enzyme located on the membrane surface of* Leishmania*, and this drug had leishmanicidal activity against* Leishmania donovani* intracellular amastigotes in a dose-dependent manner.

In their study, Nilforoushzadeh et al. 2008 [26] reported that omeprazole (40 mg) plus intramuscular meglumine antimoniate (30 mg/kg/day) showed similar clinical cure presented by meglumine antimoniate (60 mg/kg/day), being it of 89% and 93%, respectively. Moreover, omeprazole (40 mg) plus intramuscular meglumine antimoniate (30 mg/kg/day) showed greater clinical cure rate than meglumine antimoniate (30 mg/kg/day), being the cure rates of 89% and 80%, respectively.

The combination omeprazole plus meglumine antimoniate was well tolerated and the authors reported no side effects, thus it may be a clinically useful alternative likely efficacious for CL treatment.

### 4.8. Cryotherapy

Cryotherapy is a therapeutic modality recommended by the World Health Organization (WHO) for the treatment of CL. According to WHO, it is a recommended treatment regimen for Old World CL, combined or not with intralesional antimonial [4].

Above all, some studies showed that the combination of cryotherapy with intralesional pentavalent antimonial is more effective than the antimonial alone [11, 76].

The three articles included in this review, conducted by Van-Thiel et al. (2010) [29], Meymandi et al. (2011) [10] and Farajzadeh et al. (2015) [32], presented a lower cure rate for the combination of cryotherapy and intralesional sodium stibogluconate or for the combination with meglumine antimoniate.

Some variables should be taken into consideration for the performance of cryotherapy, which may directly influence the efficacy of the treatment, such as the size of the lesion and the frequency of the cryotherapy sessions. Papules smaller than or equal to 1 cm, responded more quickly to cryotherapy than lesions larger than 1 cm. According to Ranawaka et al. (2011) [77], for smaller papules the cure rate was 90.5% and for the ones larger than 1 cm, it was 64.28%.

The frequency of sessions also seems to play an important role in the effectiveness of cryotherapy. When performed weekly, cure rates were high (equal or greater than 90%), either alone or in combination with pentavalent antimonials [8, 77]. Application at longer time intervals may result in lower cure rates. Soto et al. (2013) [78] performed only two sessions of cryotherapy at intervals greater than 1 week and obtained a low cure rate (20%).

Another important fact to consider before the application of cryotherapy is the phototype of skin. In patients with phototype V, for example, depigmentation may occur. It is also necessary to investigate the tendency of keloid formation [77].

Cryotherapy is a clinically useful alternative and has few, but not serious, adverse effects. It has a high cure rate when considering the size of the lesion and the frequency of the sessions.

### 4.9. Ketoconazole

Ketoconazole is an antifungal that interferes with the biosynthesis of ergosterol, an important cell membrane constituent, essential for the viability and survival of fungi and trypanosomatids. The target of Ketoconazole is the C14*α*-demethylase and, thus, it interferes with the dimethylation of the sterol and, consequently, inhibits the synthesis of ergosterol [79].

Oral ketoconazole alone has been tested for the treatment of CL for several years and has shown different cure rates [80–83]. In this review, we included the study of El-Sayed and Anwar (2010) [21], which tested the combination of intralesional sodium stibogluconate and oral ketoconazole (600 mg/day). This combination was more effective than the ketoconazole and sodium stibogluconate alone.

Saenz et al. (1990) [80], using ketoconazole alone (600 mg/day), obtained a cure rate of 73% and Salamanpour et al. (2001) [82] found a cure rate of 89% in the treatment with ketoconazole (600 mg/day) alone.

Possibly the species is a determinant factor in the efficacy of ketoconazole. WHO recommends ketoconazole (600 mg/day) as the treatment regimen for CL in the New World, specifically when the etiologic agent is* Leishmania mexicana*, although there are reports of its efficacy in other species [4]. El-Sayed and Anwar (2010) [21] did not identify the species in their study. Saenz et al. (1990) [80] also did not identify it, but their study was conducted in Panama. Salmanpour et al. (2001) [82] cited that the patients had Old World CL. Ramanathan et al. (2011) [83] demonstrated efficacy in the treatment of CL by* Leishmania panamensis*. With respect to ketoconazole resistance, Andrade-Neto et al. (2012) [84] demonstrated that* Leishmania amazonensis* can up-regulate the C-14 demethylase in response to ketoconazole, which may contribute to its resistance to this drug.

Oral administration of ketoconazole combined with intralesional sodium stibogluconate for the treatment of CL is shown acceptable risk with specialized monitoring and no serious adverse effects and in administration are reported.

### 4.10. Pentoxifylline

Pentoxifylline is a derivative of dimethylxanthine classified as a vasodilator agent. It exerts effects on different cell types, such as reduction of the expression of adhesion molecules with ICAM- 1 in keratinocytes and E-selectin in endothelial cells, inhibition of TNF-*α* synthesis, IL-1 and IL-6 and antifibrinolytic effects [14, 85].

In particular, pentoxifylline may potentiate the action of pentavalent antimonials primarily by two mechanisms: increase in the expression of the inducible nitric oxide synthase (iNOS) and, consequently, increase in the production of nitric oxide, and anti-TNF-*α* action [86, 87]. Brito et al. (2014) [88] observed that patients treated with pentoxifylline (400 mg - 3 times per day) combined with meglumine antimoniate (20 mg^5+^/kg/day) had greater TNF-*α* suppression than those treated with meglumine antimoniate alone (20 mg^5+^/kg/day), and cure rates were higher in the combined group than in the second group.

Machado et al. (2007) [15] demonstrated in their study that the combination of meglumine antimoniate (20 mg^5+^/kg/day) and pentoxifylline (400 mg - 3 times per day) potentiated the effect of the meglumine antimoniate, since the combination resulted in 82% of cure in patients with mucosal leishmaniasis, while meglumine antimoniate (20 mg^5+^/kg/day) alone had a cure rate of 41.6%. Sadeghian and Nilforoushzadeh (2006) [17], in which this same combination was tested to treat cutaneous leishmaniasis (in endemic area for* Leishmania major*) and resulted in 81.3% cure versus 51.6% for meglumine antimoniate alone. In contrast, at the same conditions in the cited studies, Brito et al. (2017) [31] reported a cure rate of 43% for a combination of pentoxifylline and meglumine antimoniate to treat cutaneous leishmaniasis caused by* Leishmania braziliensis*, as divergences in cure rates may be related to intrinsic characteristics of each patient to pentoxifylline, and the specie of* Leishmania*.

The anti-TNF-*α* action of pentoxifylline makes its use interesting, mainly in cases of mucosal and/or treatment-refractory leishmaniasis, since this cytokine is the main responsible for mucosal damage. There have been reports of success in the combination of pentoxifylline and meglumine antimoniate in the treatment of treatment-refractory cases [14] and with high production of TNF-*α* [89] or recurrent cases [90].

For Lessa et al. (2001) [14], the efficacy of the combination pentoxifylline and meglumine antimoniate should make it the second choice in the treatment, since the administration is oral and has fewer adverse effects than amphotericin B.

The efficacy of pentoxifylline in combination with meglumine antimoniate in the treatment of mucocutaneous leishmaniasis, even in cases refractory to conventional and/or recurrent treatment, added to few and not severe effects, makes this combination a good therapeutic alternative clinically useful for treatment of mucocutaneous leishmaniasis. However to treat cutaneous leishmaniasis with this combination it is necessary to take into account the species involved, since in cases caused by* Leishmania braziliensis* this combination showed not efficacious and not useful.

### 4.11. Clinical Implications

The first choice drugs for the treatment of cutaneous or mucocutaneous leishmaniasis do not always show the expected result, so the association of these conventional drugs with others drugs or modalities of therapy, such as local therapies have good cure rates, often higher than those of the drugs of choice, and few adverse effects. Above all, the combination with immunomodulators seems to be promising, even with limited numbers study and patient it was surprisingly effective, revealing higher efficacy and few adverse effects. In the case of combination with local application therapies, the diameter of the lesion appears to be an important factor for successful treatment. In addition to efficacy, many combinations are easy to administer by the patient and without the need for specialized monitoring, what represents an advantage for use in more isolated communities.

### 4.12. Strengths and Limitations of the Study

This systematic review has gone through many steps in its development. The precision in publications' search was guaranteed by two databases. Publications' identification criteria were monitored and discussed in many steps of the research to guarantee robustness and rigor of the findings. Special care was also taken for the identification of the MeSH terms, which were decided by many researchers and by consensus, providing good sensitivity and specificity. The publications' findings were organized and detailed in four tables for better clarity and quality of data. Concerning the limitations, we identified that only four of the 16 articles included in the review highlighted the limitations topic (Llanos Cuentas et al. 2010; Khatami et al. 2013; Farajzadeh et al. 2015; Brito et al. 2017). Other limitations were the inclusion of only two databases, with publications merely in English comprising the period from 12/2004 to 6/2017. The considerable heterogeneity between the articles included, mainly due to the significant variation of both the substances used and the research regions, made it impossible to analyze the data more precisely, for example through meta-analysis. Despite these limitations, we believe the results can contribute positively for the treatment of cutaneous leishmaniasis and mucocutaneous leishmaniasis.

## 5. Conclusion

The combination of pentavalent antimonial drugs with other drugs seems to be a good alternative to conventional treatment, since they presented good cure rates, often higher than those of the drugs of choice, and few adverse effects. Therefore, this type of combination deserves to be investigated in more detail by clinical trials and prospective studies with more robust population sample to reinforce the effectiveness and safety that this alternative treatment provides to the patient.

## Figures and Tables

**Table 1:** Baseline characteristics of clinical trials included in the analysis of combinations for the treatment of tegumentary leishmaniasis.

**Study source**	**Area, Country**	**Study design**	**Period of study**	**Age range, mean (years)**	**Gender**	**Clinical form**	**Patients enrolled**	**Leishmaniasis diagnosis**	**Statistics**
Almeida et al., 2005	Bahia, Brazil	Open-label clinical trial	NR	14-25, 18	Male 60% Female 40%	Cutaneous	5	Clinical and Laboratory (skin test, and isolation in culture)	No

Arevalo et al., 2007	Lima, Peru	Comparative study; Randomized Controlled Trial	8/2005–10/2005	18-87, 34.9	Male 55% Female 45%	Cutaneous	20	Clinical and Laboratory (microscopy, culture, and/or PCR, and Montenegro skin test)	Yes

Brito et al., 2017	Bahia, Brazil	Randomized -controlled trial	12/2010–10/2013	18-62	Predominance of male	Cutaneous	162	Clinical and laboratorial (Leishmania skin test, and/or histopathology, culture and PCR)	Yes

El-Sayed & Anwar, 2010	Sanaa, Yemen	Comparative study; Randomized Controlled Trial	6/2006–6/2007	12-50, 23.5	Male 53.3% Female 46.7%	Cutaneous	30	Clinical and Laboratory (smear for amastigote and tissue culture)	Yes

Farajzafeh et al., 2015	Iran	Randomized clinical trial	2011-2012	2-60, 18.52	Male 34 Female 36 NR 10	Cutaneous	80	Laboratory (smear microscopy)	Yes

Firooz et al., 2006	Razavi, Iran	Multicenter Study; Randomized Controlled Trial	8/2004-/2005	12-60, 27.0	Male 44.5% Female 55.5%	Cutaneous	119	Laboratory (smear or culture)	Yes

Khatami et al., 2013	Kashan, Iran	Randomized Controlled Clinical Trial	9/200–4/2010	12-60, 28.8	Male 47% Female 53%	Cutaneous	83	Laboratory (smear or culture)	Yes

Llanos Cuentas et al., 2010	Cusco, Peru	Randomized Controlled Trial	8/2004 – 6/2005	18-59	Male 96% Female 4%	Mucosal	48	Laboratory (microscopy, PCR or *in vitro* culture)	Yes

Machado et al., 2007	Bahia, Brazil	Randomized Controlled Trial	NR	18-65	Male 83% Female 17%	Mucosal	23	Laboratory (Intradermal skin test, parasite isolation by culture, and/or histopathological)	Yes

Meymandi et al., 2011	Kerman, Iran	Comparative Study; Randomized Controlled Trial	11/2007-8/2009	7-60	Male 46.6% Female 53.4%	Cutaneous	191	Laboratory (smear or skin biopsy)	Yes

Miranda-Verastegui et al., 2005	Lima, Peru	Randomized Controlled Trial	2/2001 – 8/2002	1-78	Male 57.5% Female 42.5%	Cutaneous	40	Laboratory (aspiration, smear, biopsy, culture, and/or PCR)	Yes

Miranda-Verastegui et al., 2009	Lima and Cusco, Peru	Comparative Study; Randomized Controlled Trial	12/2005-6/2006	4-52	Male 77.5% Female 22.5%	Cutaneous	80	Laboratory (smear microscopy, culture or PCR)	Yes

Nascimento et al., 2010	Minas Gerais, Brazil	Randomized Controlled Trial	10/2004–10/2006	18-59, 26.4	Male 63.6% Female 36.4%	Cutaneous	44	Laboratory (microscopy identification in biopsied tissue)	Yes

Nilforoushzadeh et al., 2007	Isfahan, Iran	Randomized Controlled Trial	NR	7-70	Male 67.7% Female 32.3%	Cutaneous	90	Laboratory (smear microscopy)	Yes

Nilforoushzadeh et al., 2008	Tehran, Iran	Comparative Study; Randomized Controlled Trial	NR	7-70	Male 71.0% Female 29.0%	Cutaneous	124	Laboratory (smear microscopy)	Yes

Van Thiel et al., 2010	Northern Afghanistan,	Clinical Trial	6/2005-11/2005	NR	Dutch Troops	Cutaneous	163	Laboratory (smear microscopy, culture, and PCR)	Yes

NR, not reported; PCR, polymerase chain reaction.

**Table 2:** Clinic, therapeutic, and epidemiological characteristics of clinical trials included in the study.

**Study source**	***Leishmania* species**	**Treatment**	**Patients at the end**	**Previous treatment**	**Clinically healed**	**Therapy failed**	**Relapse or Recurrence**
Almeida et al., 2005	*L. braziliensis*	G1 (MA + GM-CSF)	5	Yes	100% (before 120 days AS) Follow-up: 12 months AH	0% (12 months AH)	0% (12 months AH)

Arevalo et al., 2007	*Leishmania *spp.	G1 (IM)	6	No	0% Follow-up: 3 months AS	67% (20 days AS)	33% (3 months AS)
		G2 (MA)	7	No	57% (3 months AS) Follow-up: 3 months AS	43% (3 months AS)	0% (3 months AS)
		G3 (MA + IM)	7	No	100% (3 months AS) Follow-up: 3 months AS	0% (3 months AS)	0% (3 months AS)

Brito et al., 2017	*L. braziliensis* (62% of cases)	G1 (MA + PE)	82	NR	45% (6 months AE)	55% (6 months AE)	NR
		G2 (MA + placebo)	82		43% (6 months AE)	57% (6 months AE)	NR

El-Sayed & Anwar, 2010	NR	G1 (il SSG)	10 (12 lesions)	No	50%/58.3% (12 weeks AS; patients/lesions) Follow-up: 6 months AE	50%/41.7% (12 weeks AS; patients/lesions)	NR
		G2 (il SSG + im SSG)	10 (15 lesions)	No	90%/93.3% (12 weeks AS; patients/lesions) Follow-up: 6 months AE	10%/6.7% (12 weeks AS; patients/lesions)	NR
		G3 (il SSG + KE)	10 (13 lesions)	No	90%/92.3% (12 weeks AS; patients/lesions) Follow-up: 6 months AE	10%/7.7% (12 weeks AS; patients/lesions)	NR

Farajzadeh et al., 2015	NR	G1 (Terbinafine + cryotherapy)	40	No (within the past 90 days)	37.5% complete (28 days AS) 10 partial cure^#^	15 (28 days AS)	NR
		G2 (MA + cryotherapy)	40	No	52.5% (21 days AS) 7 partial cure^#^	12 (21 days AS)	NR

Firooz et al., 2006 *∗∗*	*L. tropica*	G1 (MA + IM)	42	No	18.6% (4 weeks AS) 44.1% (8 weeks AS) 50.8% (20 weeks AS) Follow-up: 16 weeks AE	49.2% (20 weeks AS)	3.1% (16 weeks AS)
		G2 (MA + placebo)	47	No	30.0% (4 weeks AS) 48.3% (8 weeks AS) 53.3% (20 weeks AS) Follow-up: 16 weeks AE	46.7% (20 weeks AS)	8.1% (16 weeks AS)

Khatami et al., 2013	*L. major*	G1 (il MA)	23 (40 lesions)	No	12.5% (6 weeks AS) lesions 40.0% (10 weeks AS) lesions Follow-up: 5 months AE	65.0% (6 weeks AS) 42.5% (10 weeks AS)	0% (5 months AE)
		G2 (il MA + non-silver PD)	21 (46 lesions)	No	6.5% (6 weeks AS) lesions 42.2% (10 weeks AS) lesions Follow-up: 5 months AE	80.4% (6 weeks AS) 55.6% (10 weeks AS)	0% (5 months AE)
		G3 (il MA + silver PD)	29 (55 lesions)	No	12.7 (6 weeks AS) lesions 36.4 (10 weeks AS) lesions Follow-up: 5 months AE	74.6% (6 weeks AS) 49.1% (10 weeks AS)	3.4% (5 months AE)

Llanos Cuentas et al., 2010	*L. braziliensis*	G1 (SSG + placebo)	12	No (within the past 30 days); 22.0% had received previously	50% (84 days AS) 100% (336 days AS) Follow-up: 336 days AS	25% (168 days AS) 0% (336 days AS)	8% (336 days AS)
		G2 (SSG + (LEISH-F1 + MPL-SE))	LEISH-F1 5*µ*g = 11		59% (84 days AS) 94% (336 days AS) Follow-up: 336 days AS	13% (168 days AS) 6% (336 days AS)	0% (336 days AS)
			LEISH-F1 10 *µ*g = 12				
			LEISH-F1 20 *µ*g = 11				

Machado et al., 2007	*L. braziliensis*	G1 (MA + placebo)	12	No*∗*	41.6% (90 days AS) Follow-up: 150 days AS	42% (150 days AS)	0% (2 years AE
		G2 (MA + PE)	11	No*∗*	82% (90 days AS) Follow-up: 150 days AS	0% (150 days AS)	0% (2 years AE)

Meymandi et al., 2011	*L. tropica*	G1 (C0_2_ laser)	80	No	56.8% (2 weeks AS) 67.6% (6 weeks AS) 44.4% (12 weeks AS) 93.7% (89/95 lesions) Follow-up: 16 weeks AS	NR	NR
		G2 (il MA + cryotherapy)	80	No	15.8% (2 week AS) 57.5% (6 week AS) 38.2% (12 week AS) 78% (74/95) lesions) Follow-up: 16 weeks AS	NR	NR

Miranda-Verastegui et al., 2005	*L. peruviana* *L. braziliensis*	G1 (MA + IM)	18 (35 lesions)	Yes	6% (20 days AE) 50% (1 month AE) 61% (2 months AE) 72% (3 months AE) 72% (6 months AE) 72% (12 months AE) Follow-up: 12 months AE	27.8% (12 months AE)	NR
		G2 (MA + Vehicle)	20 (40 lesions)	Yes	5% (20 days AE) 15% (1 month AE) 25% (2 months AE) 35% (3 months AE) 50% (6 months AE) 75% (12 months AE) Follow-up: 12 months AE	25% (12 months AE)	NR

Miranda-Verastegui et al., 2009	*L. peruviana* *L. guyanensis* *L. braziliensis*	G1 (SSG + vehicle cream)	36	No	17.5% (20 days AS) 33% (1 month AS) 30% (2 months AS) 60% (3 months AS) 63% (6 months AS) 58% (9 months AS) 53% (12 months AS) Follow-up: 12 months AS	41.7% (12 months AS)	NR
		G2 (SSG + IM)	39	No	5% (20 days AS) 43% (1 month AS) 60% (2 months AS) 78% (3 months AS) 75% (6 months AS) 75% (9 months AS) 75% (12 months AS) Follow-up: 12 months AS	23.1% (12 months AS)	NR

Nascimento et al., 2010	*Leishmania spp.*	G1 (MA + (LEISH-F1 + MPL-SE))	LEISH-F1 5*µ*g = 9	No	80% (84 days AS) Follow-up: 336 days AS	24% (84 days AS)	4% (84 days AS)
			LEISH-F1 10*µ*g = 8				
			LEISH-F1 20*µ*g = 8				
		G2 (MA +MPL-SE)	8	No	50% (84 days AS) Follow-up: 336 days AS	50% (84 days AS)	NR
		G3 (MA +Saline)	8	No	38% (84 days AS) Follow-up: 336 days AS	62% (84 days AS)	38% (84 days AS)

Nilforoushzadeh et al., 2007	*Leishmania *spp.	G1 (il MA + topical honey)	33	No	51.1% (6 weeks AS) Follow-up: 4 months AS	48.9% (6 weeks AS)	NR
		G2 (il MA)	35	No	71.1% (6 weeks AS) Follow-up: 4 months AS	28.9% (6 weeks AS)	NR

Nilforoushzadeh et al., 2008	*L. tropica* *L. major*	G1 (MA 60 mg/kg/day + placebo)	43	No	93% (12 weeks AS) Follow-up: 12 weeks AS	7% (12 weeks AS)	NR
		G2 (MA 30 mg/kg/day + OM)	36	No	89% (12 weeks AS) Follow-up: 12 weeks AS	11% (12 weeks AS)	NR
		G3 (MA 30 mg/kg/day + placebo)	45	No	80% (12 weeks AS) Follow-up: 12 weeks AS	20% (12 weeks AS)	NR

Van Thiel et al., 2010	*L. major*	G1 (il SSG)	118	No	55.1% (6 months AE) Follow-up: 6 months AE	20.3% (6 months AE)	15.3% (6 months AE)
		G2 (il SSG + cryotherapy)	45	No	66.7% (6 months AE) Follow-up: 6 months AE	13.3% (6 months AE)	11.1% (6 months AE)

NR, not reported; G1, Group 1; G2, Group 2; G3, Group 3.

MA, meglumine antimoniate; PE, pentoxifylline; GM-CSF, granulocyte macrophage colony-stimulating factor; IM, imiquimod; il SSG, intralesional sodium stibogluconate; im SSG, intramuscular sodium stibogluconate; KE, ketoconazole; il MA (intralesional meglumine antimoniate); non-silver PD, non-silver containing polyester dressing; silver PD, silver containing polyester dressing; SSG, sodium stibogluconate; LEISH-F1, lyophilized LEISH-F1 protein; MPL-SE, adjuvant; OM, omeprazole.

AS: after the start of treatment, AE: after the end of treatment, and AH: after the healing of the lesion.

*∗*No previous treatment of mucosal leishmaniasis. Some patients had previous cutaneous leishmaniasis, but there are no references to previous treatment or not.

*∗∗*Clinical cure rate, therapy failure, and relapse or recurrence given by Firooz et al., 2006, based on the initial number of patients allocated in each group.

^#^Partial cure Farajzadeh: decrease in induration size between 25 and 75%.

**Table 3:** Description of adverse effects of combinations for the treatment of tegumentary leishmaniasis.

**Treatment **	**Side effects**	**Study source**
MA + IM	Localized pruritus, erythema and edema (77%); arthralgia, myalgia, flu-like symptoms (86%); and elevated liver enzyme levels (64%).	Arevalo et al., 2007
	Moderate pruritus and burning sensation (7.1%).	Firooz et al., 2006
	Edema (35%); itching (10%); burning (15%); pain (5%); erythema (55%).	Miranda-Verastegui et al., 2005

MA + PE	Nausea (27.3%); arthralgias (9.1%); dizziness, abdominal pain, and diarrhea (9.1%).	Machado et al., 2007
	Vomiting (2.4%); Diarrhea (1.2%); Nausea (8.6%); Headache (11%); Asthenia (3.7%); Anorexia (3.7%); Epigastralgia (3.7%); Pain (2.4%); Dizziness (2.4%); Fever (7.4%); Arthralgia (8.6%); Myalgia (13.5%)	Brito et al., 2017

MA + cryotherapy	No adverse effects were observed	Farajzadeh et al., 2015

MA + (LEISH-F1 + MPL-SE)	Local: induration (44.4 – 77.8%); erythema (11.1 – 100%); tenderness (33.3-44.4%). Systemic: headache (0-22.2%); pyrexia (0-22.2%). MA-related AEs (22.2 – 88.9%).	Nascimento et al., 2010

MA + GM-CSF	No adverse effects were observed	Almeida et al., 2005

MA + OM	NR	Nilforoushzadeh et al., 2008

il MA + silver PD	Itching and burning (35.3%); edema (33.3%).	Khatami et al., 2013

il MA + topical honey	Dermatitis to honey (3%).	Nilforoushzadeh et al., 2007

il MA + cryotherapy	Hyper pigmentation+trivial scar (18.7%); atrophic scar (7.5%); hypo pigmentation+trivial scar (18.8%).	Meymandi et al., 2011

SSG + (LEISH-F1 + MPL-SE)	Local: induration (41.7 – 75.0%); erythema (50.0 – 100.0%); tenderness (66.7 – 91.7%). Systemic: anorexia (0 – 8.3%); fatigue (0 – 8.3%); malaise (25.0%); myalgia (0 – 8.3%); headache (33.3 – 50.0%). SSG-related (100%).	Llanos Cuentas et al., 2010

SSG + IM	Swelling (30%); itching (25%); pain (12.5%); erythema (32.5%).	Miranda-Verastegui et al., 2009

il SSG + im SSG	im SSG: Pain at the injection site (100%). il SSG: Pain and swelling at the intralesional injection site (100%).	El-Sayed & Anwar, 2010

il SSG + KE	KE: No. il SSG: Pain and swelling at the intralesional injection site (100%).	El-Sayed & Anwar, 2010

il SSG + cryotherapy	Secondary infection (31%); lymphatic involvement (48.8%); pain at the injection site	Van Thiel et al., 2010

NR, not reported; G1, Group 1; G2, Group 2; G3, Group 3. MA, meglumine antimoniate; PE, pentoxifylline; GM-CSF, granulocyte macrophage colony-stimulating factor; IM, imiquimod; il SSG, intralesional sodium stibugluconate; im SSG, intramuscular sodium stibugluconate; KE, ketoconazole; il MA (intralesional meglumine antimoniate); non-silver PD, non-silver containing polyester dressing; silver PD, silver containing polyester dressing; SSG, sodium stibugluconate; LEISH-F1, lyophilized LEISH-F1 protein; MPL-SE, adjuvant; OM, omeprazole; AEs, adverse events.

**Table 4:** Conclusion on combination treatment as a new treatment of tegumentary leishmaniasis in the systematic review.

**Treatment**	**Dose**	**Route of Administration/Time**	**Time**	**Efficacy**	**Safety**	**Practice/clinical implications**	**Study source**
MA + IM	IM: Lesion ≤3 cm: 1 dose of 7.5% cream. Lesion > 3 cm: 2 doses of 7.5% cream. Each dose = 125 mg.	IM: topical - daily	20 days	Efficacious	Acceptable risk with specialized monitoring	Investigational	Arevalo et al., 2007
	MA: 20 mg/kg/day.	MA: IV - daily					
	IM: 5% cream.	IM: topical- 3 times per day	IM: 28 days	Likely efficacious	Acceptable risk with specialized monitoring	Investigational	Firooz et al., 2006
	MA: 20mg Sb^5+^/kg/day.	MA: IM daily	MA: 14 days				
	IM: 5% cream.	IM: Topical- daily.	IM: 20 days.	Efficacious	Acceptable risk without specialized monitoring	Clinically useful	Miranda- Verastegui et al., 2005
	MA: 20mg/kg/day.	MA: IM daily in children, and IV infusion in older subjects.	MA: 20 days.				

MA + PE	MA: 20mg^5+^/kg/day	MA: daily	MA: 30 days	Efficacious	Acceptable risk with specialized monitoring	Clinically useful	Machado et al., 2007
	PE: 400mg	PE: oral – 3 times daily	PE: 30 days				
	MA: 20mg^sbv^/Kg/day	MA: IV- daily	MA: 20 days	Not efficacious	Acceptable risk with specialized monitoring	Not useful	Brito et al., 2017
	PE: 400m	PE: oral- 3 times daily	PE: 20 days				

MA+ cryotherapy	Cryotherapy: freeze time (10-25 s)	Cryotherapy: on the lesion until 1-2 mm of surrounding normal tissue appeared frozen	Every two weeks	Likely efficacious	Acceptable risk without specialized monitoring	Possibly useful	Farajzadeh et al., 2015
	MA: 15 mg/kg/day	Intramuscular	Every day for 3 weeks				

MA + (LEISH-F1 + MPL-SE)	LEISH-F1: 5, 10 or 20 *μ*g + 25 *μ*g MPL-SE.	LEISH-F1: SUB – 3 times.	LEISH-F1: On day 0, 28 and 56.	Likely efficacious	Acceptable risk with specialized monitoring	Possibly useful	Nascimento et al., 2010
	MA: 10 mg/ Sb^5+^kg/day.	MA: IV – 10-days cycles followed by 11 days of rest.	MA: The first 10-days cycle on Day 0. Additional cycles on days 21, 42, and 63				

MA + GM-CSF	GM-CSF: 1-2 mL (10 *µ*g/mL).	GM-CSF: topical – 3 times per week.	GM-CSF: 3 weeks	Efficacious	Acceptable risk without specialized monitoring	Investigational	Almeida et al., 2005
	MA: 20 mg Sb^5+^/kg/day.	MA: IV – daily.	MA: 20 days				

MA + OM	MA: 30mg/kg/day	MA: IM- daily	MA: 3 weeks	Likely efficacious	Acceptable risk with specialized monitoring	Clinically useful	Nilforoushzadeh et al., 2008
	OM: 40mg	OM: oral - daily	OM: 3 weeks				

il MA + silver PD	MA: il	MA: Intradermally in each one centimeter square of a lesion until blanching occurred intralesional, once weekly.	42 days	Not efficacious	Acceptable risk with specialized monitoring	Investigational	Khatami et al., 2013
	Silver PD: on the lesion	Silver dressing: topical – once daily					

il MA + topical honey	MA: il	MA: il enough to blanch the lesion and 1 mm rim of the surrounding normal skin, once weekly.	Until complete healing or for maximum 6 weeks	Not efficacious	Insufficient evidence	Investigational	Nilforoushzadeh et al., 2007
	Honey: soaked gauze	Honey: topical – twice daily					

il MA + cryotherapy	Cryotherapy: freeze time (10 - 25 s)	Cryotherapy: on the lesion until 2-3 mm halo forms around, weekly, before IL MA.	Until complete cure or for up to 12 weeks	Likely efficacious	Acceptable risk without specialized monitoring	Possibly useful	Meymandi et al., 2011
	MA: (0.5 – 2 ml)	MA: intradermally, all directions, until the lesion had completely blanched, weekly.					

SSG + (LEISH-F1+ MPL-SE)	LEISH- F1: 5, 10 or 20 *µ*g + 25 *µ*g MPL-SE.	LEISH-F1: SUB – 3 times.	LEISH-F1: On day 0, 28 and 56.	Efficacious	Acceptable risk with specialized monitoring	Investigational	Llanos Cuentas et al., 2010
	SSG: 20mg/kg/day	SSG: IV – daily	SSG: day 0 to 27				

SSG + IM	IM: 5% cream.	IM: Topical - 3 times per week.	IM: 20 days	Efficacious	Acceptable risk without specialized monitoring	Clinically useful	Miranda- Verastegui et al., 2009
	SSG: 20 mg/kg/day	SSG: IV – daily.	SSG: 20 days				

il SSG + im SSG	il SSG (100 mg/mL), the dose varied between 0.3-3.0 mL. Maximum dose 20mg/Kg/day.	il: Infiltrated in multiple sites until complete blanching and a 1-mm wide ring of the surrounding normal skin.	il SSG on days 1, 3, 5 in one session - up to 3 cycles.	Efficacious	Acceptable risk with specialized monitoring	Possibly useful	El-Sayed & Anwar, 2010
	im SSG (a part of the dose 20mg/Kg/day already given provided to IL SSG in the same days).	im: one injection on days 1, 3, 5 - up to 3 cycles with 4 weeks interval.					

il SSG + KE	il SSG (100mg/mL), the dose varied between 0.3-3.0 mL, maximum dose 20mg/Kg/day.	il: Infiltrated in multiple sites until complete blanching and a 1-mm wide ring of the surrounding normal skin.	il: on days 1, 3, 5 in one session - up to 3 cycles.	Efficacious	Acceptable risk with specialized monitoring	Possibly useful	El-Sayed & Anwar, 2010
	KE: 200 mg.	KE: 3 times daily.	KE: 4 weeks.				

il SSG + cryotherapy	il SSG: 1-2ml.	il SSG: into margin of each lesion, all around, until blanching with cryotherapy preceding the first injection.	il SSG: 3 injections SSG with intervals of 1-3 days.	Efficacious	Acceptable risk without specialized monitoring	Clinically useful	van Thiel et al., 2010
	Cryotherapy: local with a double freeze-thaw cycle. 20 seconds for freezing cycle and thawing time between cycles of 45-90 seconds.		Cryotherapy: treatment was repeated until clinical improvement (range 1-163 days).				

MA, meglumine antimoniate; PE, pentoxifylline; GM-CSF, granulocyte macrophage colony-stimulating factor; IM, imiquimod; il SSG, intralesional sodium stibogluconate; im SSG, intramuscular sodium stibogluconate; KE, ketoconazole; il MA (intralesional meglumine antimoniate); nonsilver PD, nonsilver containing polyester dressing; silver PD, silver containing polyester dressing; SSG, sodium stibogluconate; LEISH-F1, lyophilized LEISH-F1 protein; MPL-SE, adjuvant; OM, omeprazole; IV, intravenous; IM, intramuscular; SUB, subcutaneously.
